# Microcapsules Filled with a Palm Oil-Based Alkyd as Healing Agent for Epoxy Matrix

**DOI:** 10.3390/polym8040125

**Published:** 2016-04-06

**Authors:** Nurshafiza Shahabudin, Rosiyah Yahya, Seng Neon Gan

**Affiliations:** Chemistry Department, Faculty of Science, University of Malaya, 50603 Kuala Lumpur, Malaysia; shafizashah@siswa.um.edu.my (N.S.); rosiyah@um.edu.my (R.Y.)

**Keywords:** microcapsules, renewable resources, epoxy, flexural strength, microhardness, self-healing

## Abstract

One of the approaches to prolong the service lifespan of polymeric material is the development of self-healing ability by means of embedded microcapsules containing a healing agent. In this work, poly(melamine-urea-formaldehyde) (PMUF) microcapsules containing a palm oil-based alkyd were produced by polymerization of melamine resin, urea and formaldehyde that encapsulated droplets of the suspended alkyd particles. A series of spherical and free-flowing microcapsules were obtained. The chemical properties of core and shell materials were characterized by Attenuated total reflection-Fourier transform infrared spectroscopy (ATR-FTIR) and proton nuclear magnetic resonance spectroscopy (^1^H-NMR). Differential scanning calorimetry (DSC) analysis showed a glass transition around −15 °C due to the alkyd, and a melting temperature at around 200 °C due to the shell. Thermogravimetric analysis (TGA) results showed that the core and shell thermally degraded within the temperature range of 200–600 °C. Field emission scanning electron microscope (FESEM) examination of the ruptured microcapsule showed smooth inner and rough outer surfaces of the shell. Flexural strength and microhardness (Vickers) of the cured epoxy compound were not affected with the incorporation of 1%–3% of the microcapsules. The viability of the healing reactions was demonstrated by blending small amounts of alkyd with epoxy and hardener at different ratios. The blends could readily cure to non-sticky hard solids at room temperature and the reactions could be verified by ATR-FTIR.

## 1. Introduction

The failure of a structural polymer begins from cracks within the materials. Continuous efforts are being made to overcome the damages of the cracks by integrating self-healing ability to the material. A recent review article has discussed the various types of self-healing nanocomposite materials [1]. One of the ways to achieve this objective is to store healing agents in microcapsules that are then embedded into the polymer matrix. The healing process is triggered when cracks rupture the microcapsules and release the healing agent to fill the gaps. Subsequently, the healing agent would solidify through reaction such as crosslinking with certain reactive groups of the matrix to repair the crack and prevent further damage, thus extending the lifespan of the material.

There are many recent publications on the usage of microcapsules in self-healing materials, notably in coatings, adhesives and electronic components. Various selected agents have been encapsulated, such as chlorobenzene [2], dimethyl norbornene ester and dicyclopentadiene (DCPD) [3], and norbornene dicarboximide [4]. Microcapsules of linseed oil were used to heal cracks in paint film [5] and epoxy coatings [6,7].

Urea-formaldehyde (UF) resins are widely used in adhesives, particleboard, and molded objects. It has been used in specialized applications such as the fabrication of natural fiber reinforced polymers [8]. UF has been used in the preparation of microcapsules due to several reasons. It can be crosslinked to form the shells that protect the healing agents. Nanoparticles of UF would deposit on the shell, to form a rough surface that aids in the adhesion of the microcapsules with the polymer matrix [9,10,11]. During the preparation, a low molecular weight pre-polymer was formed from the condensation of urea and formaldehyde at the initial stage. Subsequently, the pre-polymer becomes attached onto the surface of the dispersed core material and polymerize to form the shell [12,13].

Liu and co-researchers [14] have reported the modification of poly(urea formaldehyde) (PUF) resin by mixing urea with melamine-formaldehyde pre-polymer forming poly(melamine-urea-formaldehyde) (PMUF) for encapsulating 5-ethylidene-2-norbornene (ENB) and its crosslinking agent. Microcapsules with PMUF shell are more robust and easier to handle than those with PUF shell. Tong and colleagues [15] have replaced up to 12 wt % of urea with melamine in the formulation, to encapsulate an epoxy resin. They reported that the PMUF microcapsules exhibited better resistance against solvent, acid and alkali. Other researchers [16] had replaced 1%–5% of urea with a commercially available melamine resin, Cymel 303^®^, as shell materials for microcapsules containing DCPD. The microcapsules were strong enough to withstand the mixing with a viscous restorative dental resin. Nesterova and co-researchers [10] have prepared microcapsules of epoxy resins, DCPD, linseed oil and alkylglycidyl ether using both PUF and PMUF resins. They had also reported that PMUF shells were more stable and had produced higher yield.

Alkyds are polyesters that were first developed more than 85 years ago [17]. They are tough resins synthesized from a polybasic acid, a polyhydric alcohol and a vegetable oil (a triglyceride) such as soya, castor, rapeseed and linseed oils or the free fatty acids derived from the triglycerides. Alkyds are conventionally used in paints, adhesives, inks and other coatings. They have become one of the major synthetic resins in the coating industry due to their good film forming, high gloss and fast drying property [18,19]. The drying mechanism of an alkyd is attributed to air oxidation of the unsaturation in the structure. Palm oil is classified as a non-drying oil because of its low level of unsaturation, making its alkyd unable to air dry. This lack of unsaturation could be an advantage to its stability against oxidation. Palm oil-based alkyd could be made to have a certain amount of –COOH and –OH groups, which could be the reactive sites for other reactions.

Palm oil-based alkyds were found to be compatible with natural rubber and epoxidized natural rubber (ENR) and could modify the properties of rubber compounds [20,21]. Table 1 shows the effect of mixing one of the alkyd with 10% *w*/*v* ENR50 solution in toluene, where ENR50 is an epoxidized natural rubber containing 50% of the –C=C– being chemically converted to epoxide groups.

This observation has led us to the idea of using the alkyd for self-healing application in epoxy matrix. In this work, we have encapsulated the selected palm oil-based alkyd using urea, melamine resin and formaldehyde. The yield has improved and the microcapsules were more robust compared to our previous work using urea-formaldehyde [22].

## 2. Experimental Design

### 2.1. Materials

Refined, bleached and deodorized palm kernel oil (PKO) and glycerol were obtained from Emery Oleochemicals (M) Sdn. Bhd., Selangor, Malaysia. Phthalic anhydride (PA) was purchased from Hanwha Chemical (M) Sdn. Bhd., Kuala Lumpur, Malaysia, and lithium hydroxide (LiOH) was from J.T. Baker, Center Valley, PA, USA. Urea, ammonium chloride and 1-octanol were purchased from ACS Sigma-Aldrich (M) Sdn. Bhd., Selangor, Malaysia, while formaldehyde (37% aqueous) was from Systerm, Shah Alam, Malaysia. Hexamethoxymethyl melamine, Cymel 303^®^, was supplied by Cytec Industries (M) Sdn. Bhd., Selangor, Malaysia. 1,3-Dihydroxybenzol (resorcinol) and ethylene maleic anhydride copolymer (EMA, *M*_w_ = 400,000) were Riedel de-Haen reagents supplied through Sigma-Aldrich (M) Sdn. Bhd., Selangor, Malaysia. Deuterated chloroform (CDCl_3_) was from Merck Sdn. Bhd., Selangor, Malaysia. Epoxy resin used was Epikote 240, which is a low-viscosity resin with an epoxy molar mass of 185–190 g per equivalent (WPE). Epikure F205 (cycloaliphatic amine) was used as the curing agent. Both the epoxy and its curing agent were from Hexion Inc., Columbus, OH, USA. All materials were used as received.

### 2.2. Synthesis and Characterization of Palm Oil-Based Alkyd

The alkyd has an oil-length of 65% and was prepared according to the following procedure. First, 750 g PKO, 107 g glycerol and 0.7 g LiOH were charged into a reactor flask equipped with a reflux condenser, thermometer and mechanical agitator. The mixture was heated at 220 °C for about 2 h to complete the alcoholysis process. The complete conversion of the oil to monoglycerides was checked by solubility test of the product in ethanol. Heating was turned off and the temperature was allowed to drop to 180 °C before 230 g phthalic anhydride and 87 g glycerol were added. The polycondensation was carried out at 210–220 °C and the progress of reaction was monitored by acid number determination according to the recommended test method of the American Society of the International Association for Testing and Materials (ASTM) D1639-90. The reaction was stopped when the acid number has dropped to about 5% of the initial value. The product was a viscous liquid.

The alkyd was characterized by attenuated total reflectance-Fourier transform infrared (ATR-FTIR) (Perkin-Elmer Spectrum 400, Waltham, MA, USA). ^1^H-NMR spectrum was recorded on the sample dissolved in CDCl_3_ and analyzed using JNM-ECX400 II FT-NMR spectrometer, JEOL,Tokyo, Japan Viscosity of alkyd was determined by using a rheometer (Physica MCR, Anton Paar, GmbH, Graz, Austria) with a double gap (DG 26.7) accessory, equipped with a temperature regulator (Viscotherm VT, Anton Paar). Approximately 10–12 mL of alkyd was loaded into the sample holder and measurement was performed at 26 °C at constant shear rate of 100 s^−1^.

### 2.3. Synthesis of Poly(melamine-urea-formaldehyde) (PMUF) Microcapsules with Alkyd Core

Microcapsules were prepared using similar procedure [22] except that a small specified amount of melamine resin (Cymel 303^®^) was used to replace part of urea in the formulation.

### 2.4. Characterization of Microcapsules

Infrared spectra of the samples were collected from an ATR-FTIR (Perkin-Elmer Spectrum 400). The spectra of the extracted shell and core materials were compared with the neat alkyd. ^1^H-NMR spectrum of extracted core was recorded on the sample dissolved in CDCl_3_ and analyzed using JEOL JNM-ECX400 II FT-NMR spectrometer. The shell was insoluble in CDCl_3_ and thus could not be analyzed by NMR.

The total yield of the microcapsules was calculated from the weight of the product over the total weight of capsules-forming raw materials. The microcapsules could be separated into several fractions of different of sizes by sieving. The major fraction (~50%) was in the range of 300–500 μm and was selected for further characterization. The average diameter of the microcapsules was determined on data sets of more than 250 particles using images obtained from a digital microscope, equipped with measuring software (AnMo Electronics, Taipei, Taiwan) [23,24,25,26].

A known weight of the microcapsules was crushed with a pestle in a mortar. The alkyd was extracted with acetone and the insoluble shell materials were filtered, washed and dried at 70 °C for 24 h in a vacuum oven. The core content, *E*_core_, was calculated using Equation (1), where *W*_s_ refers to the weight of sample and *W*_m_ refers to the weight of the shell:

*E*_core_ = (*W*_s_ − *W*_m_)/*W*_s_ × 100, % (1)

### 2.5. Morphology of Microcapsules

The morphology of microcapsules was examined by using two different field-emission scanning electron microscopes (FESEM) (UHR-FESEM model Hitachi SU8220, Hitachi High-Tech. Corp., Tokyo, Japan and Quanta FEG 450, FEI, Oxford, UK). Samples were mounted on a single-stub sample holder and some of the microcapsules were sliced with a razor blade to facilitate examination of the interior of the microcapsules. The analysis was carried out under low vacuum using an electron acceleration voltage of 2.0 and 5.0 kV.

### 2.6. Thermal Analysis of Microcapsules

Thermal properties of the alkyd were examined using thermogravimetric analysis (TGA, Perkin-Elmer TGA 6) and differential scanning calorimetry (DSC, Mettler-Toledo DSC822e GmbH, Giessen, Germany), in a nitrogen environment at a flow rate of 20 mL·min^−1^. TGA measurement was carried from 50 to 900 °C at heating rate of 20 °C·min^−1^. DSC instrument was calibrated with an indium standard and an intercooler (HAAKE EK/90 MT, Mettler-Toledo GmbH, Giessen, Germany) was used for sub-ambient temperature. Measurement was made from −60 to 300 °C at heating rate of 10 °C·min^−1^.

### 2.7. Microcapsules Dispersion in Epoxy Matrix

The specified amounts of microcapsules were mixed with 5.0 g epoxy resin, in a small beaker. The mixture was stirred for 5 min. Then, 2.9 g of the amine hardener (58 parts per hundred parts resin (p.h.r.) was added to the mixture and carefully stirred for 5 min. As the epoxy resin started to react with the hardener, the mixture gradually thickened. It was transferred into a rectangular silicone-rubber mold with a dimension of 25 mm × 2 mm × 2 mm. The sample was cured for 8 h at ambient temperature. At that stage, the sample was not completely hardened; it was cooled in liquid nitrogen for 2 min and quickly sliced using a razor blade. The sliced pieces were then cured at 100 °C for 2 h, followed by examinations using optical microscope (OM) (optical trinocular microscope with digital camera, Optika, Ponteranica, Italy) and FESEM (Hitachi SU8220).

### 2.8. Flexural and Microhardness Tests

The samples prepared in Section 2.7 were subjected to a three-point-bend test. The control sample was prepared without the addition of microcapsules. A three-point-bend test was carried out using a universal testing machine (Shimadzu AG-X, Shimadzu Corp., Kyoto, Japan). The set up consists of two rods of 2 mm in diameter, mounted parallel with 20 mm distance. Then, 5 kN load cell were applied at a crosshead speed of 1 mm·min^−1^ until maximum stress. Each reported flexural strength was the average of five repeated samples.

Another set of samples was prepared for microhardness test using a Vickers indenter (modified from ASTM E 384-89:1990). They were prepared with a dimension of 8 mm diameter and 2 mm thickness. Test was performed on Shimadzu HMV-2 microhardness measuring machine, with test force of 98.07 mN (HV 0.01). Each sample was subjected to 3 indentations at different spots for 5 s duration per indent, and the three readings were averaged.

### 2.9. Reactions of Alkyd and Epoxy Matrix

To probe the viability of the healing reactions, small amounts of alkyd were blended with epoxy and hardener at different ratios and cured at room temperature for 24 h and the samples were then analyzed by ATR-FTIR.

## 3. Results and Discussion

### 3.1. Synthesis and Characterizations of Alkyd as Core Material

**Figure 1:**
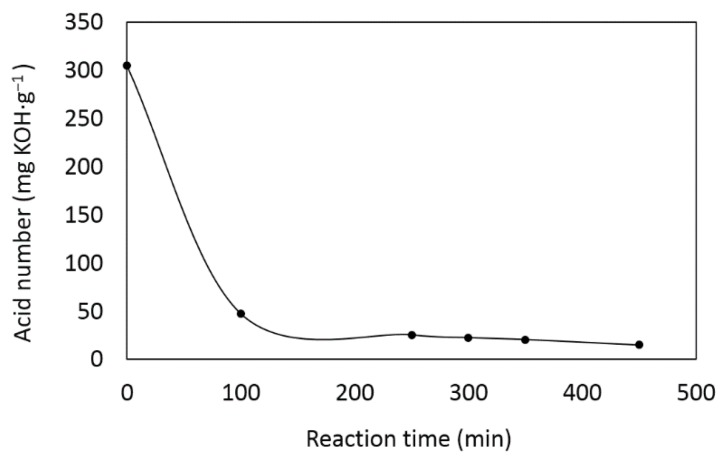
Acid number change *vs.* reaction time.

The alkyd, coded as AlkPKO65, was synthesized by alcoholysis and esterification processes. The acid number decreased with increasing reaction time as shown in Figure 1. The initial acid number of 305 mg KOH·g^−1^ was reduced to 15 mg KOH·g^−1^ in 450 min, indicating the reaction achieved >95% completion. This long-oil-length alkyd has a viscosity of 2.1 Pa·s.

**Figure 2:**
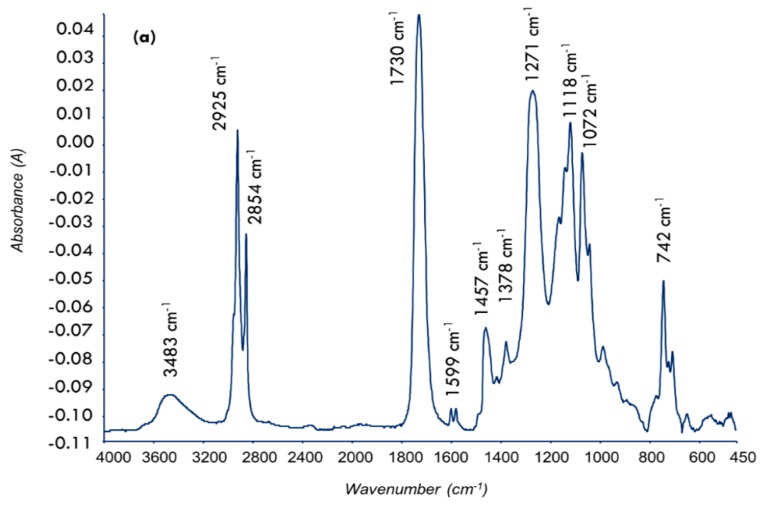

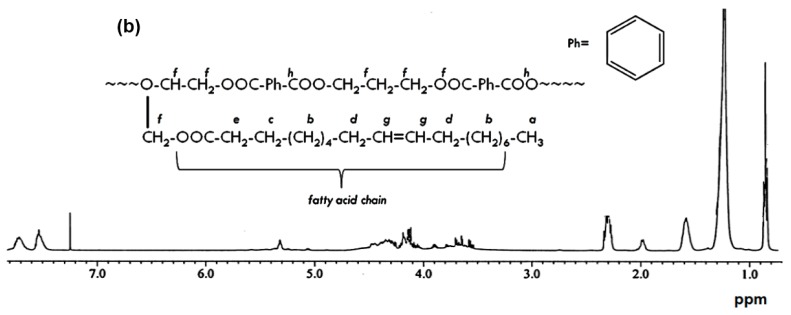
Spectroscopic characterizations of core material (alkyd AlkPKO65): (**a**) FTIR spectrum; and (**b**) ^1^H-NMR spectrum.

**Figure 3:**
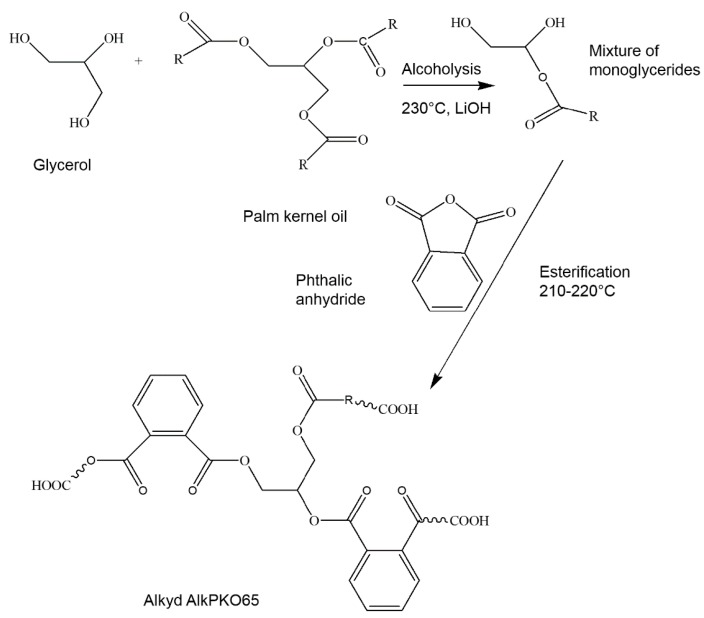
Plausible reaction in the formation of the alkyd.

The infrared spectrum of the alkyd is shown in Figure 2a. The characteristic peaks are as follows: The broad band at 3483 cm^−1^ was due to O–H stretching, sharp peaks at 2930 and 2850 cm^−1^ were attributed to C–H stretching, and strong peak at 1730 cm^−1^ was due to C=O of carboxyl groups. The small peak at 1599 cm^−1^ was attributed to the aromatic ring, and C–H and C–R bending modes were observed at 1457 and 1377 cm^−1^, respectively. The small peaks at 1072, 1122 and 1272 cm^−1^ were due to C–O groups, and the weak peak at 743 cm^−1^ was attributed to aromatic =C–H bending. The ^1^H-NMR spectrum of alkyd and the assignments of various protons were shown in Figure 2b. Table 2 summarizes the properties of the alkyd and a plausible reaction of AlkPKO65 is shown in Figure 3.

### 3.2. Synthesis of Microcapsules

**Figure 4:**
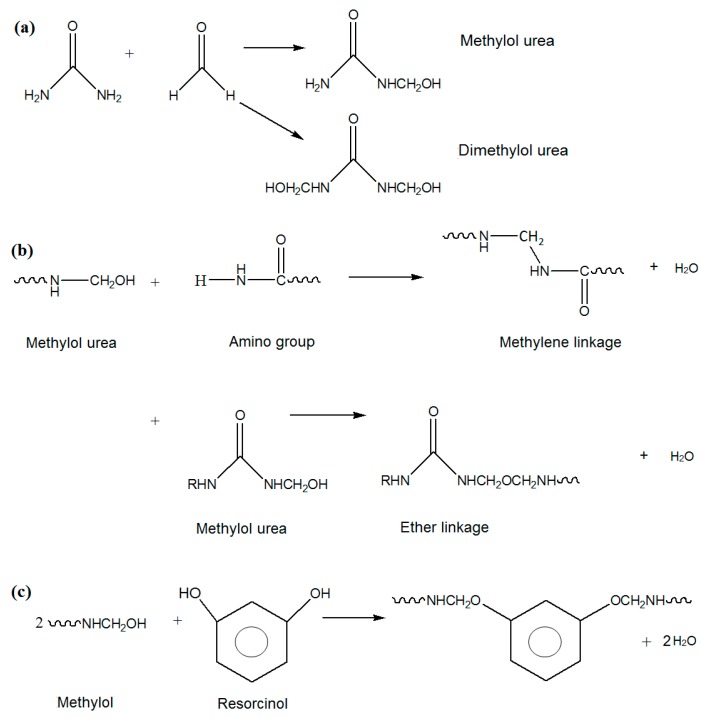
(**a**) Reactions of urea and formaldehyde to form mono- and di-methylol urea; (**b**) Reactions between methylol urea to form linkages; and (**c**) Reaction between methylol and resorcinol (as crosslinking agent).

Figure 4a shows the reactions of urea with formaldehyde to form mono- and di-methylol urea. The methylol group could react with amino group to form methylene linkage, and with other methylol group to form ether linkage (Figure 4b). In addition, it could also react with –OH groups of resorcinol as in Figure 4c.

**Figure 5:**
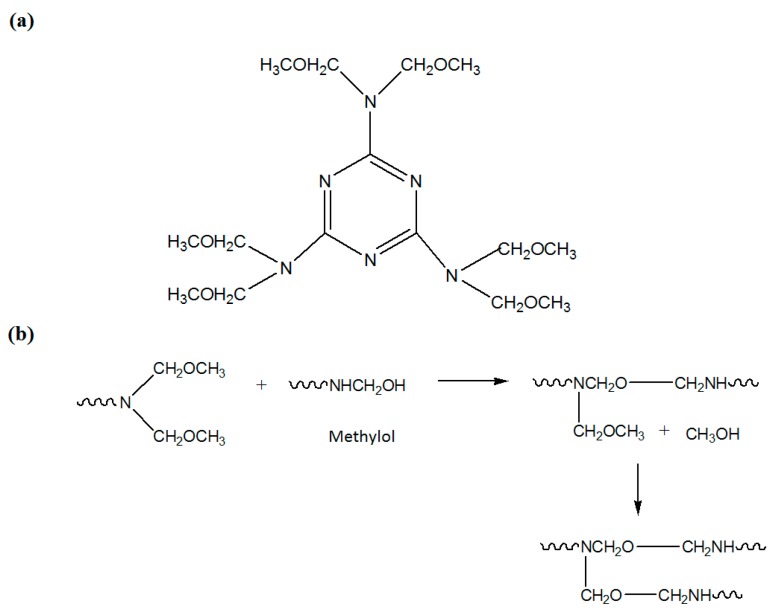

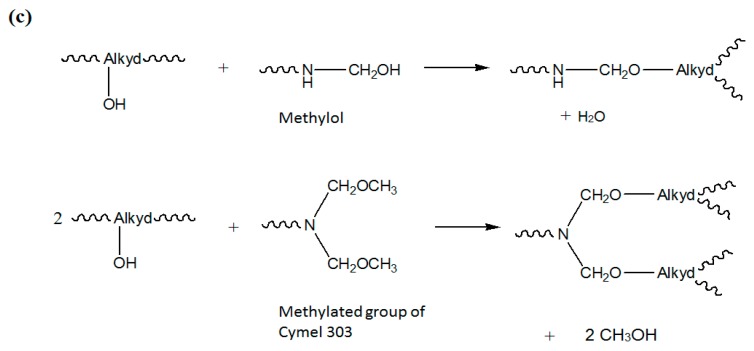
(**a**) Chemical structure of melamine resin (Cymel 303^®^); (**b**) Plausible reaction of melamine resin; and (**c**) Plausible reactions of alkyd with methylol urea and –N–CH_2_–O–CH_3_ of melamine resin.

Figure 5 shows the melamine resin, Cymel 303^®^, which has up to six methylated groups and can react with both methylol and hydroxyl groups. The methylated melamine also could react with the hydroxyl group on the surface of alkyd droplets.

### 3.3. Effect of Melamine Resin to Urea (M/U) Ratio

**Figure 6:**
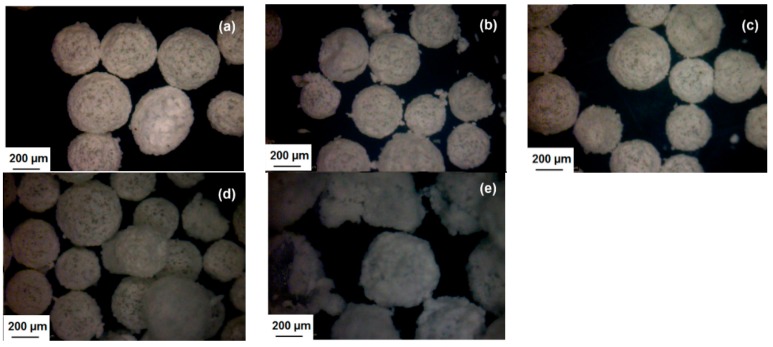
Digital microscopic images of microcapsules with increasing M/U ratio: (**a**) 0; (**b**) 0.03; (**c**) 0.06; (**d**) 0.12; and (**e**) 0.29.

With reference to the result in Table 3, sample A2, which was without melamine resin, has produced microcapsules in low yield. With the addition of a small amount of melamine resin, at melamine resin to urea (M/U) ratio of 0.03 (sample B1), the yield has increased to 65%. In addition, the microcapsules are more robust, presumably due to the crosslinking reactions of the melamine resin. However, further increase in M/U ratio to 0.06 and 0.12 in sample B2 and B3, have led to lower yield of 60% and 49%, respectively. The larger amounts of melamine resin have increased the reactions with the urea-formaldehyde pre-polymer in the aqueous medium, forming more agglomerated particles and consequently less microcapsules. Sample B4 at M/U ratio of 0.29 has led to a mixture of particles with irregular shapes whereas the other samples formed spherical and free-flowing microcapsules, as exhibited in Figure 6.

### 3.4. Spectroscopic Characterizations of Alkyd and Microcapsules

**Figure 7:**
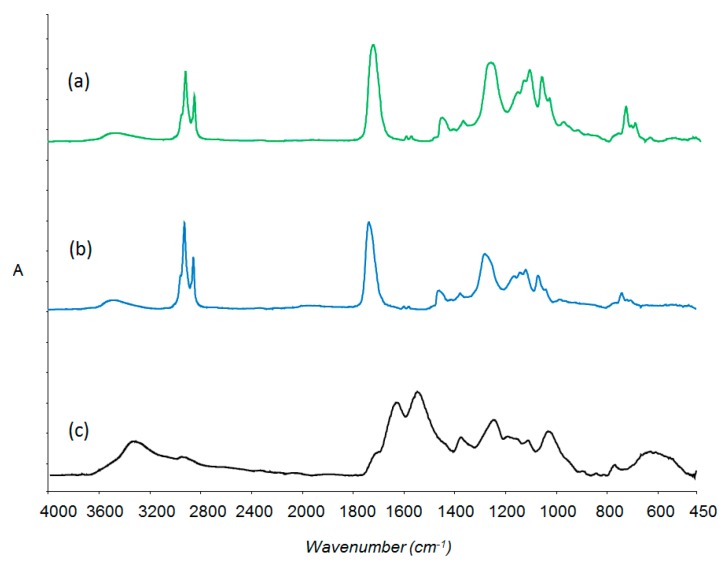
Infrared spectra of: (**a**) Neat alkyd; (**b**) Extracted core; and (**c**) PMUF shell.

Figure 7 shows the infrared spectra of: the neat alkyd (a); the extracted core (b); and shell materials (c) of sample B2. The spectrum of the extracted core shows a good match to the spectrum of the neat alkyd. Spectrum (c) shows the characteristic peaks of PMUF with peaks at 1600 and 1500 cm^−1^ correspond to NH and C–N, respectively.

**Figure 8:**
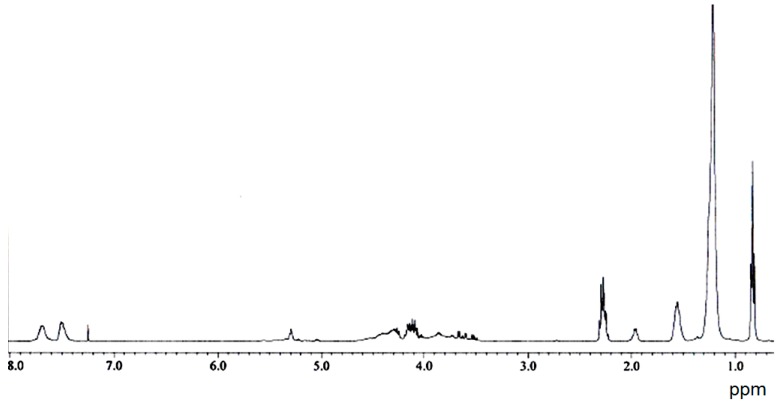
^1^H-NMR spectra of extracted core of sample B2.

The ^1^H-NMR spectrum of extracted core of B2 also showed a good match to the neat alkyd as shown in Figure 8. The shell was insoluble in CDCl_3_ and its NMR spectrum could not be measured.

### 3.5. Thermal Analysis

**Figure 9:**
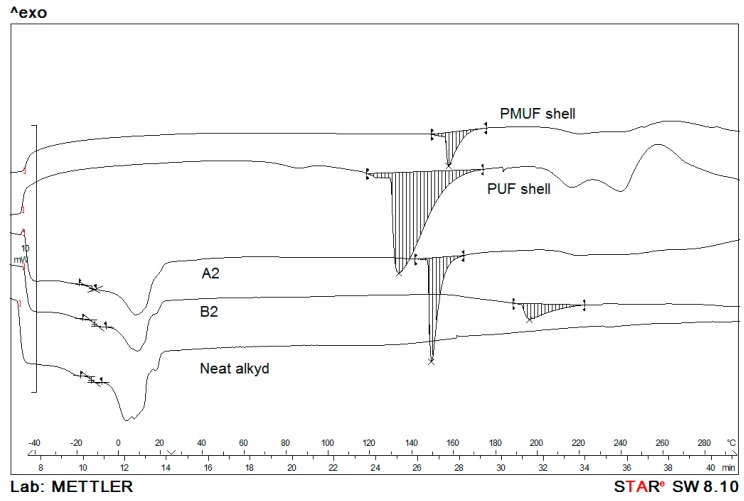
DSC thermograms of samples A2 and B2, the neat alkyd and shell materials.

Figure 9 shows the DSC thermograms of samples A2 and B2 compared to the neat alkyd and shell materials. Both samples show glass transition (*T*_g_) and melting (*T*_m_) peaks, which correspond to the *T*_g_ and *T*_m_ of the encapsulated alkyd. The *T*_g_ of A2 and B2 were −13.0 and −11.6 °C, respectively, while the *T*_g_ of the neat alkyd was −13.2 °C. The slightly higher *T*_g_ of B2 indicated the possibility of some reactions between the melamine resin with the –OH group on the surface of the alkyd droplet. Similar to the neat alkyd, A2 and B2 showed broad melting peaks area 0–15 °C. At the higher temperature range, A2 and B2 exhibited melting peaks of the shells at 148 and 192 °C, which correspond to the melting of PUF and PMUF, respectively. A very small amount of the melamine resin was used to increase the amount of crosslinking reactions to achieve a more robust shell. The shell is not 100% crosslinked. With reference to our previous paper, the microcapsules with PUF shell has a melting peak around 150 °C, the slightly higher crosslinking due to melamine resin has led to the higher melting peak observed at 192 °C for microcapsules B2.

**Figure 10:**
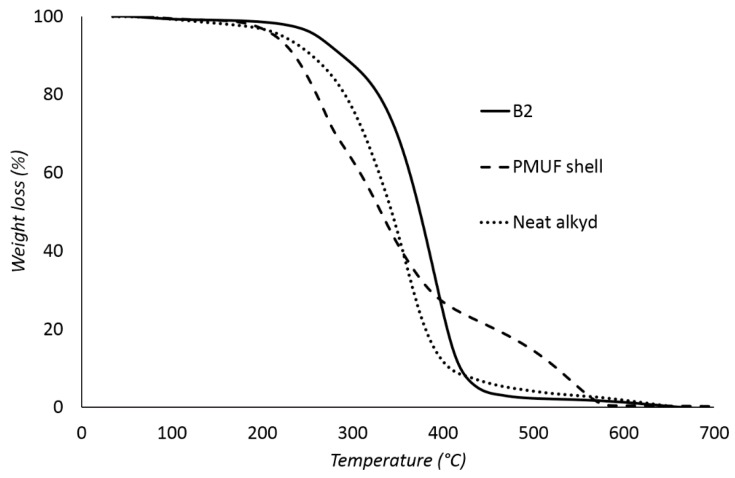
TGA thermograms of B2, neat alkyd and PMUF shell.

Figure 10 shows the thermal degradations of sample B2, the neat alkyd, and the PMUF shell. B2 was thermally stable up to 258 °C and subsequently decomposed completely within the range of 260–550 °C. Degradation of PMUF occurs around 220–300 °C, while the alkyd has started to break down around 250 °C. The thermal degradations of the core and shell have occurred in overlapping temperature ranges; consequently, TGA could not be used to determine the amount of core and shell accurately. The TGA data of the other samples are summarized in Table 4. All microcapsules were thermally stable up to ~250 °C. *T*_50%_ is the temperature at 50% weight loss and the results showed that half of the microcapsules have thermally degraded around 352 to 375 °C.

### 3.6. Morphology of Microcapsules and Epoxy Matrix

**Figure 11:**
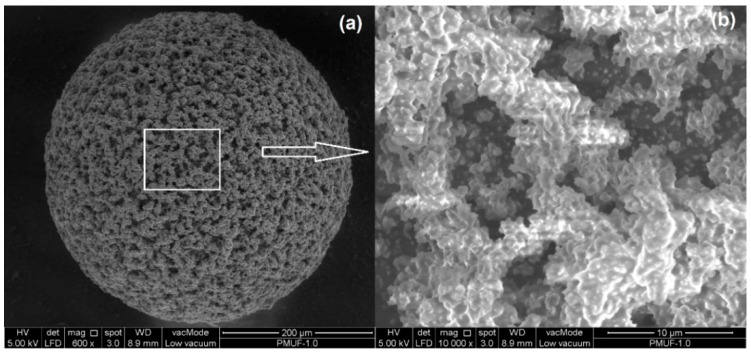
FESEM micrographs of a microcapsule at: (**a**) 500× and (**b**) 10,000× magnifications.

**Figure 12:**
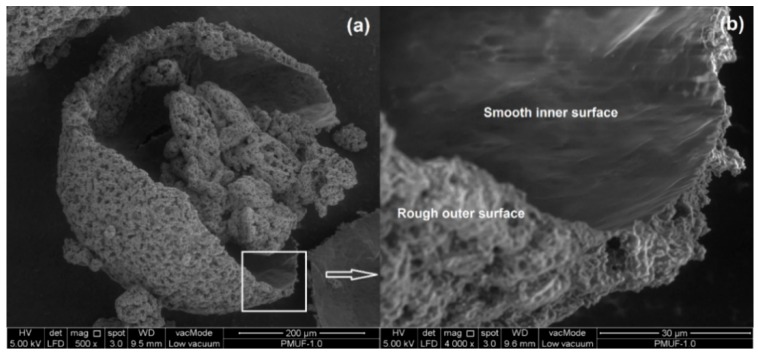
FESEM micrograph of a ruptured microcapsule at: (**a**) 500×; and (**b**) 4000× magnifications.

As shown in Figure 11a, the PMUF microcapsule was spherical in shape. Figure 11b shows the magnified region of the rough outer surface, which consisted of PMUF nanoparticles. The examination on a broken microcapsule shows that the microcapsule has smooth inner surface and rough outer surface, as shown in Figure 12.

**Figure 13:**
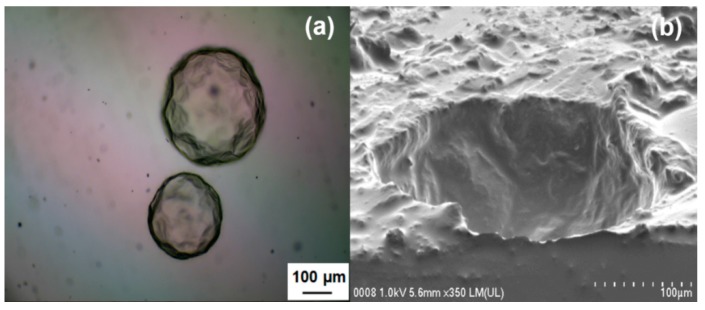
(**a**) Optical microscopic image of microcapsules embedded in the epoxy matrix; (**b**) FESEM micrograph of a sliced epoxy matrix showing a cavity previously occupied by a microcapsule.

Figure 13a shows the optical microscopic image of B2 embedded in the epoxy matrix. In Figure 13b, the FESEM micrograph shows a sliced epoxy matrix that exhibits clearly a cavity previously occupied by a microcapsule.

### 3.7. Flexural and Microhardness of Epoxy Matrix Loaded with 1%–6% Microcapsules

**Figure 14:**
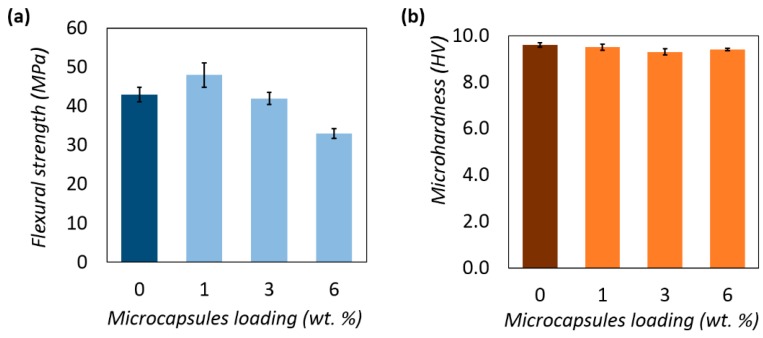
Effect of the microcapsules loading on the mechanical properties of the epoxy matrix: (**a**) Flexural strength; and (**b**) Microhardness (Vickers).

The effect of loading B2 into the epoxy matrix on flexural strength and Vickers microhardness are shown in Figure 14. The epoxy matrix without microcapsules served as the control. Incorporation of 1 and 3 wt % microcapsules did not have a noticeable effect on the flexural strength of the epoxy matrix. The flexural strength has dropped by 23% after incorporating 6 wt % of microcapsules. However, (1–6) wt % loading of microcapsules did not affected the microhardness of the epoxy matrix. Obviously, the amount and distribution of microcapsules in the polymer matrix would greatly influence the mechanical behavior of the composites and this can be optimized to achieve the best balance of self-healing and mechanical performance. It has been reported that inclusion of dispersed rubbery particles into epoxy polymer can increase their toughness without significantly diminishing the other desirable engineering properties [27,28,29]. Results of the present study show that loading of microcapsules of not more than 3 wt % did not affect the flexural strength and microhardness of the epoxy matrix.

### 3.8. Investigating the Reactions of the Alkyd Blended with Epoxy Resin and Hardener at Different Ratios

The alkyd, epoxy resin and its hardener were blended by different equivalent ratios as shown in Table 5. EA2 and EA3 were formulated with excess equivalent (Eq.) of epoxy resin. Mixing was carried out manually and all the blends were able to cure to non-sticky solid at room temperature (rt) in 24 h.

**Figure 15:**
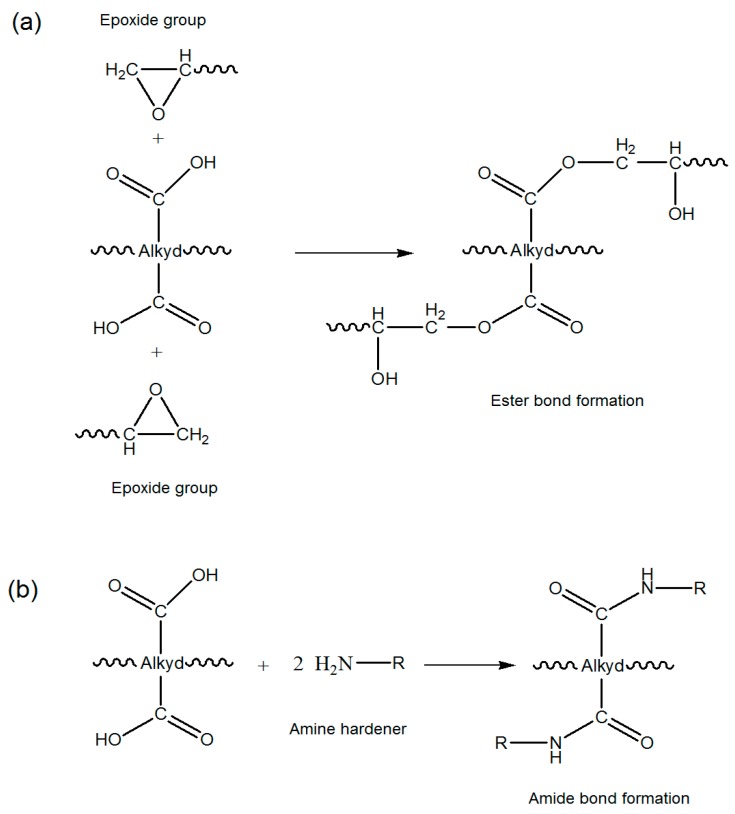
Scheme of plausible reactions of carboxylic groups of alkyd with: (**a**) Epoxy; and (**b**) Amino group.

The plausible reaction of the alkyd and the epoxy resin is shown in Figure 15. Besides that, the carboxylic acid groups of the alkyd might also react with the amino group of the hardener.

**Figure 16:**
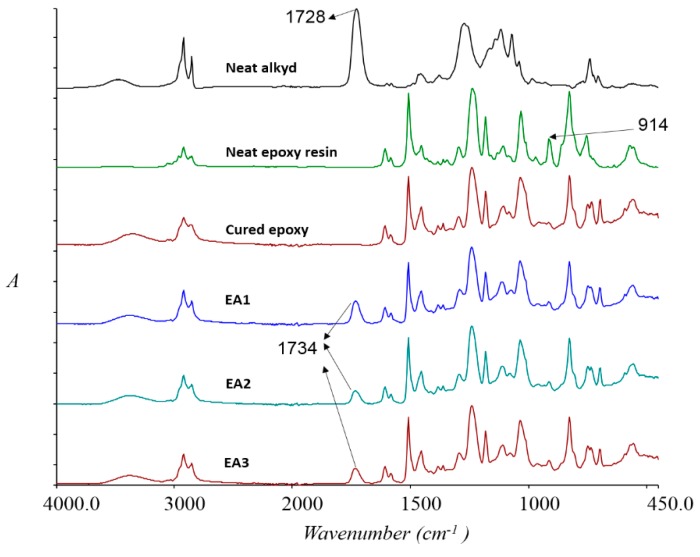
FTIR spectra of neat alkyd, epoxy resin and cured epoxy, EA1, EA2 and EA3 samples.

Figure 16 shows the FTIR spectra of neat alkyd, neat epoxy resin, and the cured samples. The spectrum of neat epoxy resin showed a strong adsorption at 2900–2800 cm^−1^ due to C–H stretching. The adsorption peaks at 1607 and 1508 cm^−1^ were attributed to C=C stretching of aromatic ring and C–C stretching of ring, respectively. The strong peaks at 1240–1030 cm^−1^ were due to C–O–C stretching of ether group. The adsorption at 914 cm^−1^ was attributed to the oxirane group. The spectra of the cured blends (EA1, EA2 and EA3) showed the peak at 914 cm^−1^ has diminished as the epoxy group was consumed in reactions. The carboxylic acid groups of alkyd at 1728 cm^−1^ has shifted to 1734 cm^−1^ in the cure samples, presumably due to conversion to ester.

## 4. Conclusions

The selected palm oil-based alkyd has been successfully encapsulated by the PMUF resin to produce free-flowing microcapsules with good yield. In comparison to PUF shell, the polyfunctional melamine resin could introduce some crosslinking reactions to improve the strength of the shell. The inner surface of the shell was smooth while the outer surface was rough. The microcapsules could be mixed into the epoxy matrix and remain embedded during the setting of the epoxy resin by its hardener. Loading of (1–3) wt % of microcapsules did not affect the flexural strength and microhardness of the epoxy matrix. The blends of small amounts of alkyd into epoxy and amine hardener mixture could readily cured at room temperature. The viable reaction is supported by the FTIR results.

## Figures and Tables

**Table 1:** The observed effects of reacting alkyd with ENR50 at different weight ratios in toluene at room temperature (around 27 °C).

Details	Sample 1	Sample 2	Sample 3
Ratio of alkyd:ENR50	1:9	1:1	9:1
Initial observation	Clear solution	Clear solution	Clear solution
After 4 h	Turned viscous	Observable gel	Phase separation
Upon removal of toluene	Sticky mass	Elastic solid	Brittle solid

**Table 2:** Properties of alkyd AlkPKO65.

Characteristic	AlkPKO65
Oil length (%)	65
Acid number (mg KOH·g^−1^)	15
Viscosity (Pa·s)	2.14

**Table 3:** Characterization data for alkyd microencapsulation.

Sample	M/U Ratio	M (g)	U (g)	Yield (%)	Core-Content (wt %)	Mean Diameter (µm)	Description of Microcapsules (MCs)
A2	0	0	2.50	40	89.9 (0.5)	403 (56)	Spherical, free-flowing
B1	0.03	0.08	2.49	65	94.8 (0.3)	383 (56)	Spherical, free-flowing
B2	0.06	0.16	2.47	60	92.0 (1.3)	380 (60)	Spherical, free-flowing
B3	0.12	0.30	2.45	49	91.9 (0.4)	384 (55)	Spherical, free-flowing
B4	0.29	0.70	2.40	–	–	–	Mixture of irregular shapes

M: Melamine resin; U: Urea. The same amount of formaldehyde was used in all cases. Values in parentheses are the standard deviation.

**Table 4:** TGA data of microcapsules, core and shell.

Sample	*T*_d_ Onset (°C)	*T*_50%_ (°C)
A2	250	352
B1	250	375
B2	258	375
B3	245	369
Alkyd	250	342
PUF shell	220	310
PMUF shell	220	331

*T*_d_: Onset degradation temperature.

**Table 5:** Reactions of alkyd, epoxy and amine hardener in different blends.

Sample	Eq. wt Ratio of Epoxy/Amine/Alkyd	Epoxy/Alkyd wt Ratio	Epoxy (g)	Amine (g)	Alkyd (g)	After 24 h at rt
Control	1/1/0	100/0	1	0.58	0	Cured, solid
EA1	1/0.8/0.2	100/39	1	0.44	0.39	Cured, solid
EA2	1/0.8/0.1	100/20	1	0.44	0.20	Cured, solid
EA3	1/0.7/0.1	100/20	1	0.39	0.20	Cured, solid
